# Supplementary material for: Patterns of Intron Gain and Loss in Fungi
Source: PLoS Biol. 2004 Nov 30;2(12):e422. doi: 10.1371/journal.pbio.0020422 (PMC532390; doi:10.1371/journal.pbio.0020422)
Supplement: Table S1 — Also available at http://genes.mit.edu/NielsenEtAl/. (4.3 MB ZIP). [file pbio.0020422.st001.zip › NielsenEtAl/html/821.html]

AN3853.1.NCU01272.1.MG02440.1.FG05902.1


```
 CLUSTAL W (1.82) Multiple Sequence Alignments - Introns Inserted


Sequence 1: NCU01272.1	1012 aa
Sequence 2: FG05902.1	1004 aa
Sequence 3: MG02440.1	988 aa
Sequence 4: AN3853.1	1049 aa
Alignment Length: 1058 aa
Number Identitical Residues: 440 aa
Alignment Score (without introns) 23594


MG02440.1 	MLR-------------RAALGARG------------AGRSYATVTQQFPQPGERLHGFTM
NCU01272.1	MLR-------------NATKGA---------------ARRAVTELSQYPKPGEKLHGFTL
FG05902.1 	MLR-------------NAAAG----------------ARKAVTELSQFPKPGEKLHGFTL
AN3853.1  	MLRSYLHLGRHRTPAFRQPLGRLLRPTASILQYAQSRTLASVSSLESLPEVGDQLHGFTV
          	***:    .   :.: . . *    .:::  . :.:     .:  .. *: *::*****:

MG02440.1 	KRIKHVPELKLTALELQHDKTGAEHLHIARNDSNNVFSIGFKTNPPDDTGLPHILEHTTL
NCU01272.1	LRSKHVPELELTALHLQHDKTGAEHLHIARDDSNNVFSIGFKTNPPDDTGVPHILEHTTL
FG05902.1 	VRSKHVPELELTALHLQHDKTGADYLHIARDDSNNVFSIGFKTNPPDDTGIPHILEHTTL
AN3853.1  	QEKKQVPELHLTAIRLRHDKTHADYLHIAREDKNNVFGIGFKTNPPDATGVPHILEHTTL
          	 . *:****.***:.*:**** *::*****:*.****.********* **:*********

MG02440.1 	CGSEK2YPIRDPFFKMLPRTLSNFMNAFTASDHTFYPFATTNAKDFQNLMSVYMDATLNP
NCU01272.1	CGSQK2YPIRDPFFKMLPRTLSNFMNAFTASDHTFYPFATTNAQDFKNLMSVYLDATLHP
FG05902.1 	CGSEK2YPIRDPFFKMLPRTLSNFMNAFTASDHTFYPFATTNAQDFKNLMSVYLDSTLHP
AN3853.1  	CGSEK2YPIRDPFFKMLPRSLSNFMNAFTSSDHTMYPFATTNQQDFQNLLSVYLDATMHP
          	***:* *************:*********:****:******* :**:**:***:*:*::*

MG02440.1 	LLKKTDFWQEGWRVGPENPEALAAAGAEATPEDKRLVFKGVVYNEMKGQMSDASYLFYIR
NCU01272.1	LLKETDFTQEGWRIGPENPQALVAAEGNAKPEDRKLVFKGVVYNEMKGQMSDAAYLFWIR
FG05902.1 	LLKKSDFTQEGWRIGPENP----LAEDEAS---KKLVFKGVVYNEMKGQMSDAGYLYYIR
AN3853.1  	LLKEEDFRQEGWRLGPEDPRAIQTQEGNLKP--EDILFKGVVYNEMKGQMSDANYLYWIR
          	***: ** *****:***:* :      : ..  . ::**************** **::**

MG02440.1 	FQDHIFPDINNSGGDPQKITDLTYEQLKRFHADHYHPSNAKLFTYGDMPLADHLQEIDAQ
NCU01272.1	FQDHIFPDIHNSGGDPQKITDLTYQQLKKFHADHYHPSNAKVFTYGDMPLADHLKEIGAQ
FG05902.1 	FHDHIFPDINNSGGDPQKITDLTYEQLQKFHAEHYHPSNAKVFTYGDMPLIDHLKQVDTQ
AN3853.1  	FQESIFPAINNSGGDPQHITDLTHKQLVEFSKKNYNPSNAKIITYGDMPLADHLKQVGGV
          	*:: *** *:*******:*****::** .*  .:*:*****::******* ***:::.  

MG02440.1 	LSAFNKLSSE-----------------------------DVAK0SSVSWVLGDTGDIVES
NCU01272.1	LDVFEKIRADVAHHSPIDLSSGPREVKLYGPIDPLVDANKQFK~TSVSWVLGETNNVVES
FG05902.1 	LQAFEKIQGDKQVHEPVTLN-GPKEVTLYGPLDPLVDQDRQYK~TSVSWIMGDTTDVLES
AN3853.1  	LNDFSKGAVDTTVKLPIELR-GPINVTVPGPIDTFVSEDRQFK~TSTSWYMGDITDTVET
          	*. *.*   :     .     .. . .  .. ..  . .   * :*.** :*:  : :*:

MG02440.1 	FSLSLISALLMDGYGSPMYKGLIESGLGIAWTPNSGYDNSAKDGIFSIGLTGLQEQDVPK
NCU01272.1	FSLALISALLMDGYGSPLYKGLIESGLGTDWSPNTGYDSSGKLGIFSIGLSGVQEEDVPK
FG05902.1 	FSLALLSTLLMDGYGSPLYRGLIEAGMGADWSPNAGYDSSAKKGIFSIGLTGVQEGDVPK
AN3853.1  	FSAGILSSLLLDGYGSPMYKALIESGLGSSFTPNTGLDTSGKIPIFSIGVTGVSEEQAPR
          	** .::*:**:******:*:.***:*:*  ::**:* *.*.*  *****::*:.* :.*:

MG02440.1 	VKTEVQRILREACEQGFKRSKIDGYLHQIELGLKHKTANFGMSLLQRVKPKWFNGVDVFD
NCU01272.1	VKAKVQEILRSMRDKGFERSKIDGYLHQLELGLKHKTANFGMSLLHRLKPKWFVGVDPFD
FG05902.1 	LKEKVQQILRDARNKGFDKTKIDGSLHQLELSLKHKTANFGFSMLNRLKPKWFNGVDPFD
AN3853.1  	VKEEIQRVLQETLQRGFNDEKVQGFLHQLELALRHKTANFGLGVIQKTFTSWFNGSDPMK
          	:* ::*.:*:.  ::**.  *::* ***:**.*:*******:.::::  ..** * * :.

MG02440.1 	TLAWNETIEAFENKMAEGGYLEGLMDRYLLNDNTLTFTMTPSADFGAELGKEEETRLAAK
NCU01272.1	SLAWNDTIAAFETELAKGGYLEGLIDKYLINDNTLSFTMAPSPTFSQELAQEEETRLSTK
FG05902.1 	SLAWNDTINGFQAKMAEGNYLEGLIDKYLLNDNTLTFTMAPSTTYGEDLVKEEQERLSTR
AN3853.1  	ELAWNEVINAFKSRYEKGGYLEALMQKYLINDNCLTFTMVGTPSFNKELDDKEMARKEKK
          	 ****:.* .*: .  :*.***.*:::**:*** *:***. :. :. :* .:*  *   :

MG02440.1 	ISEVRESLGGEAEAQKALEERELQLLAEQSKSTSEDLSCLPTVRVSDIPRRKEPAVVRHD
NCU01272.1	ISEVVKAAGSEEEARAALEARELKLLAEQSKTNTEDLGCLPSVHVKDIPRQKDSVILRHD
FG05902.1 	IQAAIKEAGSEEKARKHFEKQEQELLVEQNKTNTEDLGCLPTVHVKDIPRSKEAVVVRDE
AN3853.1  	FEQLTQQHGSVEKAVTELAKAELQLLEVQEKAQHADLSCLPSLRVEDISRQKEHKPVRES
          	:.   :  *.  :*   :   * :**  *.*:   **.***:::*.**.* *:   :*..

MG02440.1 	ETDGFRTMWHEAPTNGLTYFRSINTFENLPQDLREMIPLFTDAINRLGTKDLTMEELEDR
NCU01272.1	NTARVKTQWHEAPTNGLTYFRAINQLENLPDELRSLIPLFTDSIMRLGTKDMTMEQLEDL
FG05902.1 	NANGTKIQWHEAPTNGLTYFRAINTLENLPDELRELVPLFTDSIMRLGTKDLNMEQLEDL
AN3853.1  	KVEGTDIVWREAPTNGLTYFQAVNAFADLPDDLRLLLPLFNDAIMRLGTPTRTMEQWEDL
          	:.      *:**********:::* : :**::** ::***.*:* ****   .**: ** 

MG02440.1 	IKLLTGGNLSVGYHAASSPNDFTQASEGLTFSGMVLDRNVQEMYELLRLLVLETNFDSPD
NCU01272.1	IKLKTGG-VSVGYHSASHPTDFTRATEGLMFSGMALDRHVPTMFDLLRKLVVETDFDSPQ
FG05902.1 	IKLKTGG-VSVGYHCTPSPTDFHAASEGIIFTGMALDHNVPVMFDIIQKLVLGTDFDSPE
AN3853.1  	IKLKTGG-VSTSNFHTTSPTEMGKYTEGLQFSGFALDKNVPDMLEILTALVTETDFTSPS
          	*** *** :*.. . :. *.::   :**: *:*:.**::*  * :::  **  *:* **.

MG02440.1 	AVSHIRQLLKASTEEAINQIADSGHSYARSAAEAGLSTKNHYNEQVGGLSQIKLLTSLAG
NCU01272.1	AAQQIRQLLQASADGVVNDIASSGHAYARRAAESGLTWDSFLKEQVSGLSQVKLVTSLAS
FG05902.1 	AALRIRQLLQASADGVVNDIASTGHRFAMGSAESGLTRSAWLRQQVSGLSQVQLVTSLAS
AN3853.1  	APAMIQELLRLTTNGALDAVAGTGHRYALNAAAAGLSRSFWAQEQTSGLAQLQATANLLR
          	*   *::**: ::: .:: :*.:** :*  :* :**: .   .:*..**:*::  :.*  

MG02440.1 	RPES--DQLEDVIGKLKTIQRLAFNAD-SMRTFITCGSESVQQNQTALSKFIGTLPRNNS
NCU01272.1	RPES--DPLEDVIAKLKQIQQFALAG--NLRTAITCDSGSVSDNAKALLNFVNSLPSE-A
FG05902.1 	RPET--DKLEDVISKLKQIQNLALVGG-NLRTAITCGSESVAANGASLQNFVGNLSRD-P
AN3853.1  	DAETSPERLAELIEKLRLIQSFAISKTSGLRVRLVCEPASSTQNESVLQRWVTGLPKVPS
          	 .*::.: * ::* **: ** :*:   :.:*. :.* . *   *   * .::  *.   .

MG02440.1 	VNLSSTGQTAQSFARDVKAFYPLPYQVYYGALAVPTSSYTSKDSAPLLILSQMLTHRHLH
NCU01272.1	VTFPSR--GPPNFTRDIKTFYPLPYQVYYGALALPTASYTASVNAPLQILSQLLTHKHLH
FG05902.1 	LNLKNP--SPRQLPKDSKTFYPLPYQVYYGGLSLPTTSYTSAEGAPLQILSQLLTHKHLH
AN3853.1  	PTSQPQ--RFDLSTPSKKAFYDLPYKVYYSGLALPTVPFTHSSSATLSVLSQLLTHNYLH
          	 .           . . *:** ***:***..*::** .:*   .*.* :***:***.:**

MG02440.1 	HEIREKGGAYGGGAYARSLEGIFGFYSYRDPNPVNTLKIMRNAGRWAVDKQWTDRDLEEA
NCU01272.1	HEIREKGGAYGGGSYARPLDGIFGFYSYRDPNPVNTLKIMRNAGQWAVDKEWTDRDLEDA
FG05902.1 	HEIREKGGAYGGGAYSRALDGLFGFYSYRDPNPQNTLSIMRNAGQWAVDKKWSDRDLEEA
AN3853.1  	PEIREKGGAYGAGASNGPVKGLFAFTSYRDPNPANTLKVFKNSGVFARDRAWSDREINEA
          	 **********.*:   .:.*:*.* ******* ***.:::*:* :* *: *:**::::*

MG02440.1 	KISVFKSVDAPKDINAEGMGQFLYGVTEEMAHERRARLLDVTREQIQEVANKYVVESLDK
NCU01272.1	KISVFQGVDAPKAVNEEGMAQFLYGITDEMKQKRREELLDVTKDQVREVAQEYVVKALNN
FG05902.1 	KISVFQGVDAPKSVNQEGMGRFLSGITEEMKQKKREQFLDVTKDQVREAAQRYLVDGLAK
AN3853.1  	KLGIFQGLDAPVSVDEEGSRYFLNGITHEMDQRWREQVLDVTAKDVNEVAQTFLVDGTRR
          	*:.:*:.:***  :: **   ** *:*.** :. * ..**** .::.*.*: ::*..  .

MG02440.1 	QAERVAFLGQKADWVDGS-WATKEMNVD------------
NCU01272.1	GSERVVFLGEKRDWVDKS-WAVKEMDINGST---------
FG05902.1 	GEGRVAFLGEKQAWVDGE-WQIREMDVKGAE---------
AN3853.1  	S---VCLLGEKKDWAESEGWEVRKLSMNPNGSNIPSGDAA
          	    * :**:*  *.: ..*  :::.:.   :. .:..::
```
